# Cardiovascular disease risk: it is complicated, but race and ethnicity are key, a Bayesian network analysis

**DOI:** 10.3389/fpubh.2024.1364730

**Published:** 2024-06-10

**Authors:** Nicole P. Bowles, Yimin He, Yueng-hsiang Huang, Eric C. Stecker, Azizi Seixas, Saurabh S. Thosar

**Affiliations:** ^1^Oregon Institute of Occupational Health Sciences, Oregon Health & Science University, Portland, OR, United States; ^2^Department of Psychology, University of Georgia, Athens, GA, United States; ^3^Knight Cardiovascular Institute, Oregon Health & Science University, Portland, OR, United States; ^4^Psychiatry and Behavioral Sciences, University of Miami, Miami, FL, United States; ^5^OHSU-PSU School of Public Health, Oregon Health & Science University, Portland, OR, United States; ^6^School of Nursing, Oregon Health & Science University, Portland, OR, United States

**Keywords:** mixed graphical model, NHANES, ASCVD, social determinants, cadmium, lead

## Abstract

**Background:**

Cardiovascular diseases are the leading cause of morbidity and mortality in the United States. Despite the complexity of cardiovascular disease etiology, we do not fully comprehend the interactions between non-modifiable factors (e.g., age, sex, and race) and modifiable risk factors (e.g., health behaviors and occupational exposures).

**Objective:**

We examined proximal and distal drivers of cardiovascular disease and elucidated the interactions between modifiable and non-modifiable risk factors.

**Methods:**

We used a machine learning approach on four cohorts (2005–2012) of the National Health and Nutrition Examination Survey data to examine the effects of risk factors on cardiovascular risk quantified by the Framingham Risk Score (FRS) and the Pooled Cohort Equations (PCE). We estimated a network of risk factors, computed their strength centrality, closeness, and betweenness centrality, and computed a Bayesian network embodied in a directed acyclic graph.

**Results:**

In addition to traditional factors such as body mass index and physical activity, race and ethnicity and exposure to heavy metals are the most adjacent drivers of PCE. In addition to the factors directly affecting PCE, sleep complaints had an immediate adverse effect on FRS. Exposure to heavy metals is the link between race and ethnicity and FRS.

**Conclusion:**

Heavy metal exposures and race/ethnicity have similar proximal effects on cardiovascular disease risk as traditional clinical and lifestyle risk factors, such as physical activity and body mass. Our findings support the inclusion of diverse racial and ethnic groups in all cardiovascular research and the consideration of the social environment in clinical decision-making.

## Introduction

Atherosclerotic cardiovascular disease (ASCVD) is the leading cause of death for Americans of most racial/ethnic groups in the US and has an estimated annual cost of $200 billion (1). The health burden of ASCVD is directly attributable to a lack of optimal control of risk factors (1), especially high blood pressure, high blood cholesterol level, or smoking in nearly 50% of US adults (2). Nevertheless, ASCVD mortality has declined in the 21st century, but these gains have not been uniform for all population subgroups (3, 4).

There are substantial disparities in ASCVD risk by race, ethnicity, and sex (5). These differences are often attributed to differential social and environmental exposures and health behaviors, as evident by large-scale epidemiological studies (e.g., The CARDIA study, MESA, Hispanic Community Health Study) (6). While these extensive studies have helped discover subclinical markers of ASCVD risk and helped guide ASCVD treatment at an individual level, they have stopped short of improving population-level outcomes (7). This is partly because the interactions between modifiable (e.g., health behaviors, psychosocial stressors, and occupational exposures) and non-modifiable (e.g., age, sex, and race) risk factors for ASCVD etiology are poorly understood. This gap in knowledge limits our ability to rapidly detect and predict the trajectory of ASCVD risk and develop sustainable public health policies and interventions targeting multiple exposures.

Targeting modifiable risk factors is the typical strategy for ASCVD prevention and treatment. For example, several interventions use exercise, stress reduction, or a combination of the two to reduce ASCVD risk. Such interventions assume a simple additive effect when multiple modifiable risk factors are targeted and are usually more successful at reducing ASCVD risk than interventions targeting individual risk factors (8, 9). However, the efficacy of multifactorial interventions can differ for different racial and ethnic communities (10).

Newer approaches that use machine learning can allow for ranking disease risk factors while accounting for complex characteristics of other parameters. Such techniques, including Bayesian network analysis (BNA), allow for identifying intricate patterns and relationships in large datasets, and can be beneficial to identify proximal and distal ASCVD risk markers. In this study, we used BNA and examined how multiple risk factors interact and relate to ASCVD risk using the Framingham Risk Score (FRS) and the pooled cohort equations (PCE, otherwise known as the Atherosclerotic Cardiovascular Disease Risk tool in separate models) (11, 12). We use both models here as FRS was initially recommended for estimating the 10-year risk of cardiovascular disease and has been the endpoint or focus of several clinical studies (13). The PCEs were developed in 2013 using five cohort studies and, in doing so, increased the representation of Black Americans (11) and became the recommended risk calculator for Americans between ages 40–79 years (e.g., for the prescription of statins) (14).

We estimated networks via a mixed graphical model, investigated centrality measures, and computed directed acyclic graphs using BNA to determine the causal relationships between risk factors and calculated risk using: (1) FRS for adults ages 20–79 years; (2) FRS for adults ages 40–79 years, and (3) PCE for adults between 40 and 79 years (not available in ages <40 years).

## Methods

### Study population

We utilized data from the 2005–2012 cycles of the National Health and Nutrition Examination Survey (NHANES) (15), a research program designed to assess the health and nutritional status of adults and children in the US. The ethics review board of the Centers for Disease Control and Prevention (Atlanta, Georgia) approved all protocols, and informed consent was obtained from all participants. Data were derived from a source in the public domain.^1^ Study participants were all non-pregnant adults ≥20 years old with data available for calculating their FRS and PCE (see below). To keep the data somewhat homogenous, we decided to focus on working adults; we excluded participants if they were retired, unable to work for health reasons, or were disabled. After all exclusions, we included 16,174 participants in our final analysis (Table 1). The current study used Random forest models to predict and impute missing values (16). The Random Forest (RF) model is determined based on the unbiased out-of-bag error. It offers several advantages, including applicability with many variables relative to samples, resistance to multicollinearity, suitability for non-linear trends, immunity to overfitting, and the ability to handle outliers and missing values (17). Specifics on missing data are provided in Table 1. Occupational status provided the most amount of missing data (19%).

**Table 1:** Participant and variables table.

Variable name	Count	Mean	Standard deviation	Minimum	Maximum	Variable classification	Comments
N	16,174 (8,141 females and 8,033 males)	52448.51	11461.91	31,131	71,915	n/a	n/a
Study wave	16,174	2.57	1.08	1	4	ordinal	n/a
Age	16,174	43.16	14.04	20	79	continuous	n/a
Body mass index (kg·m^−2^)	15,477	29	6.94	13.6	130.21	continuous	n/a
Occupation	13,125	3.31	1.85	1	6	categorical	occupation: 1 “managerial and professional specialty” 2 “sales” 3 “technical and administrative” 4 “service” 5 “farming, forestry, and fishing” 6 “Production, Repair”
Household income	15,475	7.63	3.15	1	13	ordinal	n/a
Education	16,158	2.47	1.11	1	4	categorical	education: 1 “less than high school” 2 “high school” 3 “some college” 4 “college grad or high”
Moderate-Vigorous physical activity	16,113	0.52	0.5	0	1	dichotomous	vigorous activity, 0 “No” 1 “Yes”
PHQ	13,870	3.19	4.32	0	27	continuous	mental health/depression, total score is based on the sum of the points in each item ranging from 0 to 27
Serum Cotinine (ng/ml)	14,717	63.55	131.24	0.01	1700	continuous	For sensitivity analysis
Blood Cadmium (ug/L)	14,833	0.54	0.65	0.11	10.8	continuous	n/a
Blood Lead (ug/dL)	14,833	1.63	1.77	0.18	61.29	continuous	n/a
Blood Mercury (ug/dL)	14,833	1.64	2.59	0.11	85.7	continuous	n/a
Sleep duration (h)	16,151	6.75	1.39	1	12	continuous	how much sleep do you get in hours
Sleep quality	16,167	1.78	0.41	1	2	dichotomous	Told doctor trouble sleeping, yes/no
Race	16,174	2.92	1.17	1	5	categorical	race: 1 “Mexican American” 2 “other Hispanic” 3 “non-Hispanic white” 4 “non-Hispanic black” 5 “other race/multi-racial”
PCE score	16,174	4.78	9.23	0	100	continuous	pool CVD equation
FRS	16,174	6.23	7.38	-10	25	continuous	Framingham risk score

### Framingham risk score

We used the FRS, a sex-specific multivariable risk factor algorithm that utilizes several established ASCVD risk factors to predict the 10-year likelihood of developing a first cardiovascular event (12). Due to the known heterogeneity in calculated risk based on different equations, we first calculated FRS for individuals 20–79 years of age and separately for individuals 40–79 years of age to compare FRS against PCE, which can only be calculated for ages 40–79 (18).

### Pooled cohort equations

We used the PCE, which is a sex- and race-specific score used in adults, to estimate the 10-year likelihood of developing a cardiovascular event, including fatal coronary artery disease. PCE included the covariates of age, treated or untreated systolic blood pressure, total cholesterol and high-density lipoprotein cholesterol levels, current smoking status (yes/no), and history of diabetes (yes/no) (11).

### Sociodemographic and health behavior/status covariates

In addition to the variables that comprise the FRS and PCE, race/ethnicity (White, African American, Hispanic American, and others), education (< 12th grade, high school diploma or GED, some college or an associate degree, and college graduate or higher), annual household income, marital status (married; living with a partner but not married; divorced, widowed, or separated; and single or never married), body mass index (BMI, kg/m^2^), physical activity level, mental health status, history of alcohol use, and average quality and quantity of sleep were also assessed (see details in the supplemental material). Participants’ longest-held occupation was grouped into seven broader categories based on previously published groups: (1) managerial and professional specialty; (2) sales; (3) technical and administrative; (4) services; (5) farming, fishing, and forestry; (6) production and repair; and (7) operators, fabricators, and laborers (19). Given the limited sample size (*n* = 4, <0.05% of the total sample), individuals who self-identified as members of the armed forces were excluded. Participants taking care of family members at home were considered service workers (20). Mental health, more specifically depressive symptoms, was assessed using the Patient Health Questionnaire (PHQ-9), (21). Alcohol use (never, lifetime, current) was considered using three questions from the alcohol use questionnaire that measured consumption in the last 12 months, consumption over a lifetime, frequency, and the number of drinks described elsewhere (22, 23). Finally, sleep was assessed using two questions to capture duration and quality. For sleep duration, a continuous variable, participants were asked, “How much sleep do you get per night (in hours or minutes?)?” For sleep quality, participants were asked yes/no, if they “Ever told [a] doctor [they] had trouble sleeping?”

### Environmental exposures

We included blood concentrations of cadmium, lead, and mercury as continuous variables as exposure measures (24).

### Statistical analyses

Descriptive statistics and secondary linear regression models were conducted using *Stata16*. All other models were conducted in R using the specific packages noted below.

#### Mixed graphical model

We used an MGM to estimate a network where edges signify conditional independent relationships among variables. The edge thickness is directly proportional to the strength of the relationship between the variables (25). We eliminated false positive edges by regularizing the model (26). We also implemented an L1 penalty by estimating a sparse inverse covariance matrix to remove trivial associations. This penalty is weighted by a parameter λ, which is more conservative than cross-validation (27). A sparse network is economical and best accounts for the covariance among variables while striving to minimize the number of edges. We used ‘mgm-package’ to estimate our MGM.

#### Centrality, closeness, and betweenness

We computed strength centrality, closeness, and betweenness centrality to signify the importance of a variable to the network to estimate causality by summing the weights of all edges connected to a node. The closeness of a node indicates its average farness to all other nodes and the betweenness indicates the number of times that a node lies on the shortest path between two other nodes. This MGM depicts associations between variables, controlling for the role of all other variables in the network, but the edges do not indicate whether X predicts Y (or vice versa) or both. In contrast, directed networks (see below) have edges with arrowheads at their tips, signifying the inferred directionality of the relationship. While they suggest a directional association, it is important to note that establishing causality requires further empirical investigation and analysis.

#### Bayesian network

Bayesian network modeling can distinguish proximal from distal causal pathways by assessing the strength and directionality of relationships between variables. Proximal causal pathways involve direct and immediate cause-and-effect connections, while distal pathways encompass indirect (with one or more mediators) or longer-term causal links. The modeling process identifies these pathways by examining how variables interact, allowing for the differentiation between immediate and more remote influences within a system.

We computed a Bayesian network, embodied in a directed acyclic graph (DAG), by running the hill-climbing algorithm provided by the R package, bnlearn (28), and as described previously by McNally and colleagues (29). We implemented a systematic step-by-step approach in the development of a Bayesian Network model for causality. First, we initiated the process by computing the structural aspect of the network. This involved adding, removing, and reversing edges to optimize a goodness-of-fit target score, specifically the Bayesian Information Criterion (BIC). Subsequently, we iteratively refined the network structure by randomly considering different candidate edges connecting various symptom pairs. Third, to ensure the stability and reliability of the resultant network, we performed a bootstrapping operation. We generate 1,000 resampled datasets during bootstrapping by randomly selecting data points from the original dataset with replacement. These resampled datasets are then used to construct a Bayesian Network independently. The process allows us to create 1,000 different Bayesian Networks, each capturing variations in the data. Afterward, we assessed the frequency of the appearance of edges in these 1,000 bootstrapped networks. An edge is retained in the final, averaged Directed Acyclic Graph (DAG) only if it appears in at least 85% of these networks.

## Results

### Regularized correlation network model for FRS between ages 20–79 years

**Figure 1:**
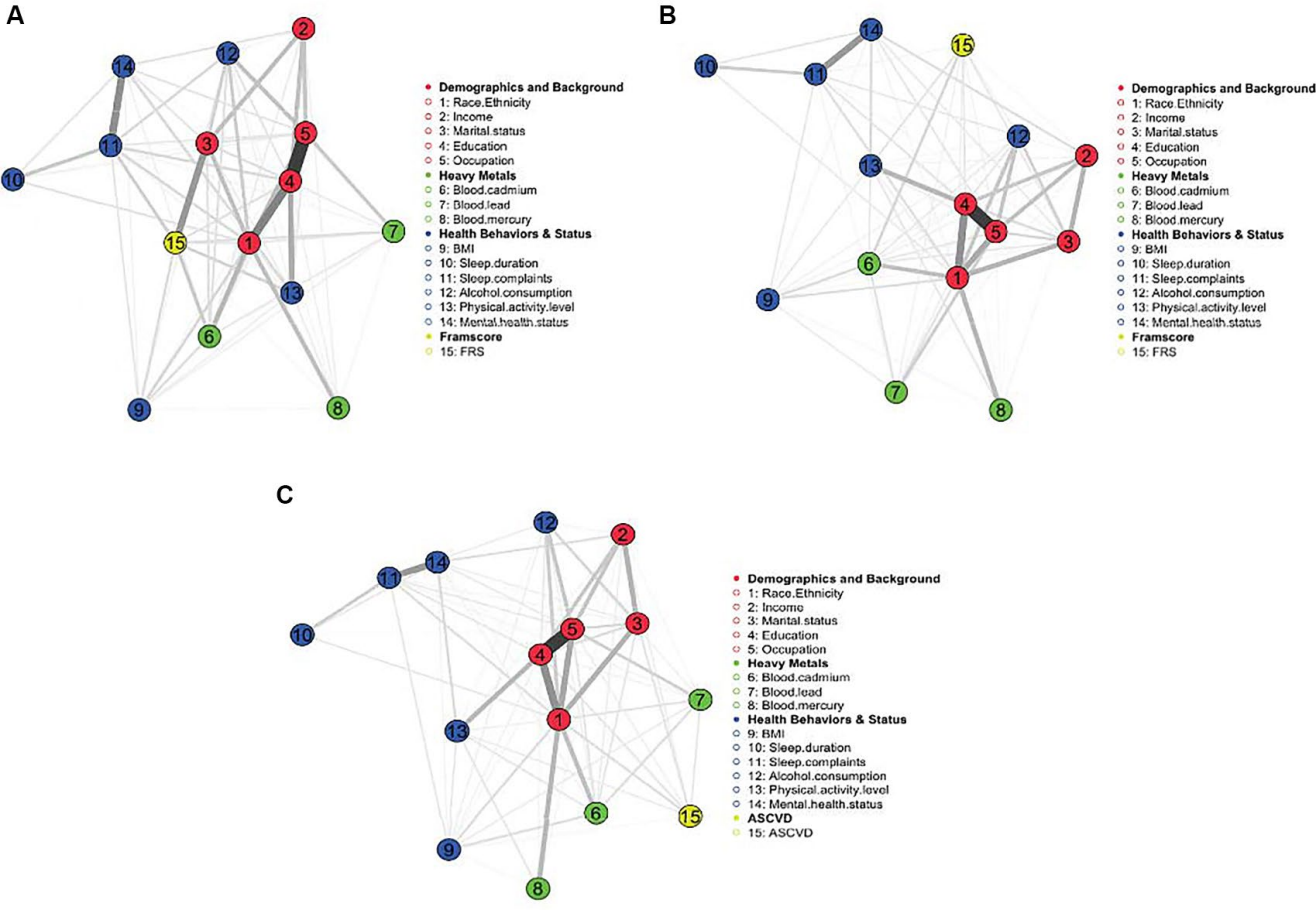
**(A–C)** MGM for FRS 20–79 years, FRS 40–79 years, and PCE (40–79 years), respectively. Demographics and background variables cluster and link with other factors, including heavy metals, health behaviors, and status. The strongest edges indicate the strongest relationships.

The MGM appears in Figure 1A. As expected, demographic and socioeconomic factors were related and clustered together. These sociodemographic factors, in turn, have relationships with other factors, including heavy metals, health behaviors and status, and FRS. Similarly, health behaviors and factors directly affected by health behaviors (e.g., BMI) and mental health cluster together, followed by the clustering of heavy metals, mainly cadmium, and mercury. Among the most robust relationships (thicker edges) are the links between education and occupation, education and race/ethnicity, sleep complaints and mental health status, education and physical activity level, marital status and FRS, and sleep complaints and mental health status. Interestingly, there appear to be strong relationships between race/ethnicity and exposure to heavy metals, especially mercury and cadmium.

**Figure 2:**
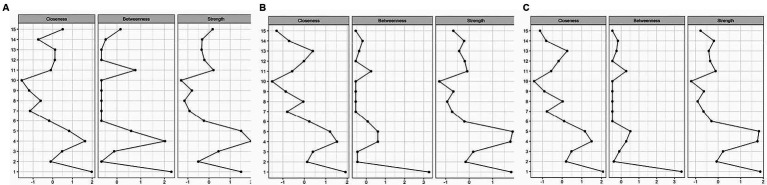
**(A–C)** Centrality plots for FRS 20–79 years, FRS 40–79 years, and PCE (40–79 years), respectively. The plot illustrates centrality metrics: strength, betweenness, and closeness between risk factors.

Figure 2A is a centrality plot that illustrates three (standardized) centrality metrics: strength, betweenness, and closeness. The determination of centrality in MGM is based on three key centrality measures. Node strength centrality assesses the importance of a node (in this case, a factor) by considering the number of connections it has to other nodes within the network. In our analysis, we calculated node strength centrality by quantifying the extent to which each factor (e.g., education, occupation, race/ethnicity, marital status, and FRS) directly influences or is influenced by other factors within the network. Betweenness centrality is an assessment of centrality in the network based on shortest paths. Closeness centrality measures how quickly a node can interact with other nodes in the network. It reflects the average distance between a node and all other nodes in the network. In our context, we computed closeness centrality to understand how ‘close’ or ‘central’ each factor is in terms of its influence on and accessibility to other factors within the network. These centrality measures help us identify which factors play pivotal roles in the network, indicating their relative importance and influence in the context of the analyzed relationships. The five factors having the greatest node strength centrality and closeness centrality were education, occupation, race/ethnicity, marital status, and FRS. Education was a highly central node in terms of the number and strength of the connections with other factors within the network. The five nodes having the highest betweenness centrality were education, occupation, race/ethnicity, sleep complaints, and FRS.

**Figure 3:**
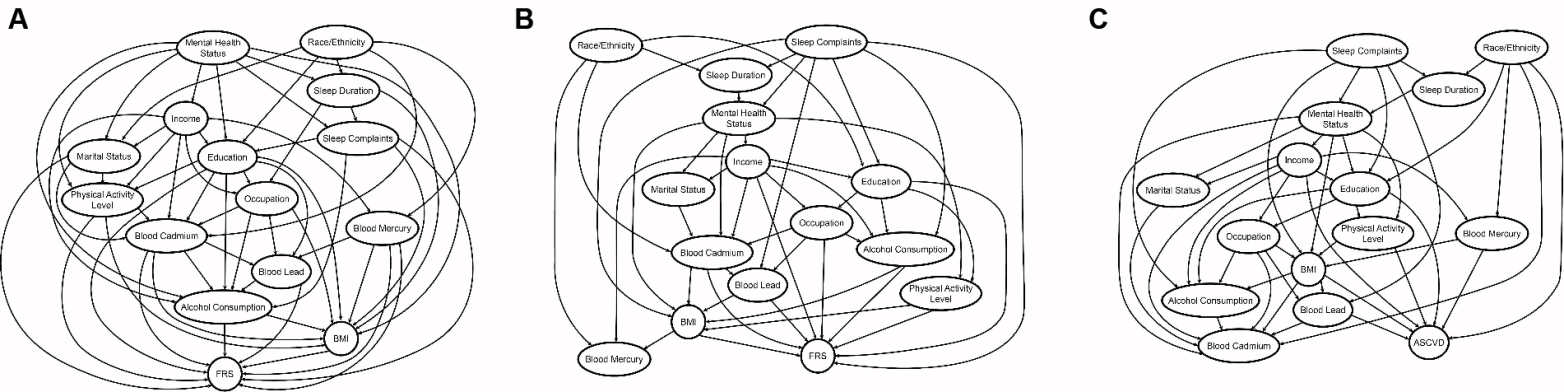
**(A–C)** The DAG’s returned by the BNA FRS 20–79 years, FRS 40–79 years, and PCE (40–79 years), respectively. The predictive priority of race/ethnicity and mental health status is the highest in FRS 20–79 years, whereas that of race/ethnicity and sleep complaints is the highest in FRS 40–79 and PCE, as these factors appear at the top of the DAG.

The DAG returned by the BNA (Figure 3A) allows us to estimate the predictive value for FRS of a factor or a group of factors. We demonstrate that the predictive (and potentially causal) priority of race/ethnicity and mental health status is the highest, as these factors appear at the top of the DAG. Race/ethnicity was found to have a meaningful association with various factors encompassing demographics, background variables, heavy metal exposure, and health-related behaviors, ultimately exerting an influence on FRS. The most direct connection between race/ethnicity and FRS was via blood cadmium and mercury levels, which directly, in turn, influence FRS. Race/ethnicity also influences education, which determines occupation, blood lead levels, and, subsequently, FRS. Mental health status also affected several sociodemographic variables, including marital status, income, and education. The most direct connection between mental health status and FRS was via alcohol consumption and BMI.

### Regularized correlation network model for FRS between ages 40–79 years

The (regularized) MGM for FRS in adults aged 40–79 appears in Figure 1B. This model’s results are nearly identical to the previous FRS model. The major exception is the relationship between marital status and FRS, which is not uncovered in this model. Additionally, FRS is very weakly related to sleep complaints and BMI, suggesting that BMI, marital status, and sleep complaints may not directly drive ASCVD risk as people get older.

The centrality plot for this model (Figure 2B) shows that the five factors having the greatest node strength centrality and closeness centrality were education, occupation, race/ethnicity, marital status, sleep complaints (strength), and blood cadmium (closeness). Race/ethnicity, occupation, and education were highly central nodes in terms of the number and strength of the connections with other factors within the network. The five nodes having the highest betweenness centrality were education, occupation, race/ethnicity, sleep complaints, and blood cadmium levels. Race/ethnicity had the most betweenness among all factors.

The updated DAG for this model (Figure 3B) also revealed the highest predictive priority of race/ethnicity and sleep complaints as these variables rise to the top of the network. In addition to the DAG above, sleep complaints directly predicted FRS and had relationships to several important risk factors, including alcohol consumption, education, mental health status, and blood mercury levels.

Given the association between cadmium and FRS, we further tested whether the source of the high blood cadmium levels was only smoking status by performing linear regression models to examine the independent association of blood cadmium and cotinine on FRS. In the initial model adjusted for race/ethnicity, increasing cadmium was associated with increased FRS (*b* = 2.92, *p* < 0.001, R^2^ = 0.07). After further adjusting for levels of cotinine, a metabolite of nicotine, levels of cadmium remained a significant independent contributor to FRS (*b* = 2.36, *p* < 0.001, R^2^ = 0.08), suggesting that the blood cadmium levels contributing to an increased FRS were due to multiple sources including, but not limited to, smoking.

### Regularized correlation network model for PCE 40–79 years

The (regularized) MGM appears in Figure 1C. Similar to the FRS models, the demographic variables cluster together and are related to other factors, including heavy metals and health behaviors. There appear to be strong relationships between race/ethnicity and exposure to heavy metals, especially mercury and cadmium.

Figure 2C is a centrality plot for PCE. The five factors having the greatest node strength centrality and closeness centrality were education, occupation, race/ethnicity, marital status, and blood cadmium. Race/ethnicity was a highly central node in terms of the number and strength of the connections with other factors within the network. The five nodes having the highest betweenness centrality were education, occupation, race/ethnicity, marital status, and sleep complaints.

The DAG for PCE (Figure 3C) shows the high predictive priority of race/ethnicity and sleep complaints. Race/ethnicity directly predicts PCE estimated risk, and also blood mercury and cadmium, among other variables. However, unlike FRS models, cadmium was not directly related to PCE estimated risk. Sleep complaints predict PCE scores via BMI and blood lead concentrations.

## Discussion

We used three approaches to understand how modifiable and non-modifiable risk factors affect cardiovascular risk scores using both the FRS and PCE calculators. Race/ethnicity was the most influential risk factor and was directly and distally related to ASCVD risk. The three main findings are: (1) Race/ethnicity had the most betweenness and closeness centrality for all three models, suggesting that these constructs have the most influence over the risk scores and other variables included in the analysis; (2) Race/ethnicity consistently demonstrated the most strength centrality and, therefore, strong connections to all other analyzed variables; and (3) Education and occupation, intimately related to race/ethnicity in this dataset, also consistently demonstrated high centrality and connections to other analyzed variables. In all three models, race/ethnicity influenced risk scores most distinctly via heavy metals, levels of mercury and cadmium in the blood in FRS 20–79 years, levels of cadmium in FRS 40–79 years, and levels of mercury in ASCVD. Finally, mental health status in FRS for ages 20–79 years, sleep complaints in FRS for ages 40–79 years, and PCE scores 40–79 years were additionally influential factors driving ASCVD risk.

Race/ethnicity and ASCVD risk: It is well-recognized that race/ethnicity is associated with ASCVD risk and that specific communities carry a disproportionate burden for cardiovascular diseases (30). Similarly, intervention effectiveness varies across different demographic groups independent of baseline risk. For instance, despite having a lower prevalence of coronary calcium than White Americans, Black Americans suffer from more adverse cardiovascular events (31). Yet, behavioral intervention effects (e.g., weight loss) are attenuated in non-White Americans compared to White Americans (10). Our BNA allowed us to account for non-linear relationships between multiple modifiable and non-modifiable factors, yet race/ethnicity continued to rise to the top as the factor driving ASCVD risk. Our findings are consistent with, and further demonstrate, similar reports that racial disparities in ASCVD risk are associated with, but not fully accounted for by socioeconomic status (e.g., education and occupation) (32). While mental health status was broadly assessed in our models by measuring depression symptoms, the proximity between mental health and race and in relation to ASCVD risk may reflect the physiological toll of chronic stress, including discrimination, job strain, and work–family conflict—hence the connections to income, occupation, and marital status (33). Similarly, mental health and connected factors are impacted by structural discrimination that results in increased exposure to neighborhood violence (34), reduced social support and access to social resources (35), as well as disparities in health care (36). Furthermore, mental health status also increases the risk of higher FRS via BMI and alcohol consumption mediators. This is in line with previous work that shows the nexus between mental health, BMI, alcohol consumption, and cardiovascular risk (37). In contrast to modifiable risk factors that focus on individual-level change, improvement of cardiovascular outcomes related to race and ethnicity will perhaps require structural changes, including health care with enhanced cultural competency training (38).

Our models demonstrate that blood levels of heavy metals also mediated the relationship between race/ethnicity and ASCVD risk. While this is a cross-sectional evaluation, heavy metal exposure could reflect environmental injustices that lead to contaminated soil, water, and food (39). It is known that heavy metals such as cadmium, lead, and mercury can increase the risk for cardiovascular morbidity and mortality (40, 41). Previous analysis of the NHANES dataset has shown that exposure to toxic metals, including cadmium, mercury, and lead, is also dependent on socioeconomic status and race (42–44). We found that even in the presence of other established risk factors, heavy metal levels are immediate predictors of ASCVD risk. Furthermore, similar to previous work (45), we found that blood cadmium levels affected ASCVD risk scores independent of smoking status.

In people between 40 and 79 years of age, sleep complaints also predicted cardiovascular risk even when not directly related. This finding is likely a function of age, as midlife (40–64 years) is an age group for rapid development of sleep disorders, including sleep apnea and insomnia, that significantly increase the risk for cardiovascular morbidity and mortality (46, 47).

The American Heart Association is promoting the concept of improving the cardiovascular health of all US citizens by 20% using eight items, including smoking cessation and regular physical activity (48). Similarly, presidents of leading cardiovascular societies across the world have called to reduce deaths from non-communicable diseases by 25% by the year 2025 (49). Based on our data, these simple approaches to controlling modifiable risk factors may be insufficient without addressing mental health, structural inequities, and practice biases. Moreover, considering these additional factors may lead to better cardiovascular disease risk calculators, as PCEs have been criticized for overestimating disease risk (50).

### Strengths and limitations

BNA offers a process-oriented approach to studying FRS and PCE estimated risk in which we begin to understand the proximal and distal drivers of ASCVD risk more clearly. BNA is remarkably suitable for modeling the complex interplay of multiple variables that influence FRS, offering a comprehensive view of how risk scores can be determined by a wide range of contributing factors beyond clinical endpoints, which can be hard to manage once they develop. In the BNA process of ascertaining the direction of each edge within bootstrapped networks, it is essential to note that the determination of edge direction is contingent on certain assumptions and conditions. Specifically, this direction is incorporated into the final network if an edge consistently ran from variable X to variable Y in at least 51% of the bootstrapped networks. However, it is essential to acknowledge that in the BNA the approach of ascertaining the direction of an edge assumes a certain level of stability in the relationships between variables under the conditions observed during the bootstrapping. Therefore, while the 51% threshold provides a measure of consistency, further sensitivity analyses and consideration of the underlying assumptions are warranted to better understand the robustness and potential limitations of the inferred edge directions in the context of the broader model.

There are other limitations worth noting. First, this study adopted a concurrent design, which cannot rule out reverse or reciprocal relations. In comparison, longitudinal, experimental, or quasi-experimental methods may provide better evidence for causality. Second, all data were from the same source, which may induce the possibility of misreporting. Future research could collect information from multiple sources to verify our findings. Race/ethnicity appears to be the key in all three analyses we conducted. However, it is necessary to note that it is included in calculating risk for the ACSVD Risk Score but not FRS.

In conclusion, using a large nationally representative dataset, we discovered that race/ethnicity is one of the foremost drivers of ASCVD risk and directly connects to all modifiable and non-modifiable risk factors. Our data strongly support the inclusion of diverse groups of individuals in all studies related to cardiovascular health and serious consideration of race/ethnicity for guideline development, diagnosis, and treatment of cardiovascular disease.

## Data availability statement

Publicly available datasets were analyzed in this study. This data can be found at: https://wwwn.cdc.gov/nchs/nhanes/continuousnhanes/default.aspx?BeginYear=2017.

## Ethics statement

The studies involving humans were approved by the ethics review board of the Centers for Disease Control and Prevention (Atlanta, Georgia). The studies were conducted in accordance with the local legislation and institutional requirements. Written informed consent for participation was not required from the participants or the participants’ legal guardians/next of kin in accordance with the national legislation and institutional requirements.

## Author contributions

NB: Conceptualization, Data curation, Formal analysis, Investigation, Methodology, Writing – original draft, Writing – review & editing. YH: Data curation, Formal analysis, Investigation, Methodology, Writing – original draft, Writing – review & editing. Y-hH: Data curation, Formal analysis, Methodology, Writing – review & editing. ES: Investigation, Methodology, Writing – review & editing. AS: Methodology, Writing – review & editing. ST: Conceptualization, Investigation, Methodology, Project administration, Supervision, Visualization, Writing – original draft, Writing – review & editing.

## Footnotes

^1^https://wwwn.cdc.gov/nchs/nhanes/continuousnhanes/default.aspx?BeginYear=2017
