# Supplementary material for: Cardiovascular disease risk: it is complicated, but race and ethnicity are key, a Bayesian network analysis
Source: Front Public Health. 2024 Jun 10;12:1364730. doi: 10.3389/fpubh.2024.1364730 (PMC11194318; doi:10.3389/fpubh.2024.1364730)
Supplement: Supplementary file 1 [file Table_1.DOCX]

**Supplemental material**

**Methods**

Details of physical activity questions: Self-reported moderate-to-vigorous physical activity (yes/no) was assessed. A "yes" could consist of participants' responses to questions regarding their transportation, household/domestic, and/or leisure-time physical activity. The self-report period for questions was different in the 2007-2012 cycles (prior week) compared to the 2005-2006 cycles (30 days). Additionally, activities around the house and work were separated in the 2005-2006 cycles but combined for the later cycles. All physical activity greater than 10 consecutive minutes was assumed to be of at least moderate intensity ^1^. Additionally, we indicated that a participant was physically active based on two questions that evaluated moderate and vigorous leisure-time physical activity: 1) "Over the past 30 days (or week in later cycles), did you do moderate activities for at least 10 minutes that caused only light sweating or a slight to moderate increase in breathing or heart rate?" and 2) "Over the past 30 days (or week in later cycles), did you do any vigorous activities for at least 10 minutes that caused heavy sweating or large increases in breathing or heart rate?"

Details of alcohol consumption criteria: Lifetime drinkers consumed ≥12 alcoholic drinks in their lifetime, whereas never drinkers consumed <12 drinks. Current drinkers consumed one or more alcoholic beverages among lifetime drinkers in the past 12 months.

Details of PHQ- 9 criteria: A 9-item screening tool that asks participants to choose 1 of 4 responses about the frequency of depressive symptoms during the previous two weeks. PHQ-9 was used in the analysis as a continuous variable.

References:

1. Ainsworth BE, Haskell WL, Whitt MC*, et al.* Compendium of physical activities: an update of activity codes and MET intensities. *Med Sci Sports Exerc* 2000;**32**:S498-504. doi: 10.1097/00005768-200009001-00009
